# The association between systemic lupus erythematosus and cognitive impairment or dementia: a meta-analysis

**DOI:** 10.3389/fimmu.2026.1795410

**Published:** 2026-06-02

**Authors:** Yanan Wang, Sai Jiang, Yicong Liu, Hejing Pan, Lin Huang, Chengping Wen, Xuanlin Li, Zhijun Xie

**Affiliations:** 1 College of Basic Medical Science, Zhejiang Chinese Medical University, Hangzhou, China; 2 Zhejiang Chinese Medical University Jinhua Research Institute, Jinhua, China

**Keywords:** cognitive impairment, dementia, meta-analysis, neuropsychiatric manifestations, systemic lupus erythematosus

## Abstract

**Background:**

Systemic lupus erythematosus (SLE) is a chronic autoimmune disease that can affect multiple organs, with neuropsychiatric manifestations, particularly cognitive impairment, being common and debilitating. However, the risk of cognitive decline and dementia in SLE patients remains debated due to methodological differences across studies.

**Objective:**

This meta-analysis aims to quantify the association between neurocognitive impairment and dementia in SLE patients by integrating data from observational studies.

**Methods:**

We systematically searched PubMed, Embase, and the Cochrane Library from inception to September 2025 for observational studies investigating the association between SLE and all-cause dementia or cognitive impairment. Study quality was assessed using the Newcastle-Ottawa Scale and the Agency for Healthcare Research and Quality checklist. Pooled effect estimates were computed using random-effects models. Heterogeneity was assessed with the I² statistic, and pre-specified subgroup analyses were performed.

**Results:**

Seven studies involving over 7 million participants were included. The meta-analysis showed a significant association between SLE and cognitive impairment, with a pooled OR (Odds Ratio) of 1.53 (95% CI: 1.26–1.85, p < 0.001), indicating a 53% higher odds. Subgroup analyses found this association in cohort studies (OR = 1.50, 95% CI: 1.20–1.88), as well as in both Asian (OR = 1.56, 95% CI: 1.08–2.26) and non-Asian populations (OR = 1.49, 95% CI: 1.36–1.64), and in both men (OR = 1.43, 95% CI: 1.13–1.80) and women (OR = 1.98, 95% CI: 1.25–3.15). Sensitivity analyses confirmed the robustness of these findings.

**Conclusion:**

SLE is associated with significantly higher odds of neurocognitive impairment, underscoring the need for integrated cognitive screening and proactive management in clinical practice.

**Systematic review registration:**

https://www.crd.york.ac.uk/PROSPERO/, identifier CRD420251148236.

## 1 Introduction

Systemic lupus erythematosus (SLE) is a chronic, multi−systemic autoimmune disease characterized by immune dysregulation, autoantibody production, complement activation, and tissue deposition of immune complexes, leading to inflammation and damage in multiple organs. Recent estimates suggest a global SLE prevalence of 43.7 per 100,000 individuals, affecting approximately 3.41 million people worldwide, with a significantly higher incidence among women (1). SLE can affect virtually any organ system, and neuropsychiatric manifestations are among the most common and clinically challenging. These manifestations affect over half (56.3%) of all SLE patients, severely impacting their quality of life and long-term prognosis (2, 3). Cognitive dysfunction is one of the most prevalent neuropsychiatric sequelae, with an estimated prevalence of 19.7%, which may be higher upon systematic assessment (3). As treatment advancements improve the life expectancy of SLE patients, managing disabling long-term outcomes such as cognitive impairment and dementia has become an increasingly pressing clinical priority (2). Several interrelated biological mechanisms may contribute to the association between SLE and cognitive decline. These include neuroinflammation caused by chronic systemic inflammation, direct neuronal damage mediated by autoantibodies such as DNRAbs (DNA-Reactive Antibodies, also known as anti-NR2 antibodies) (4) and anti-NSPA antibodies (also known as anti-ribosomal P protein autoantibodies, or anti-P) (5, 6), and cerebrovascular disease resulting from SLE-associated vasculopathy and accelerated atherosclerosis (7, 8).

While numerous observational studies have explored the relationship between SLE and cognitive impairment or dementia, their findings remain inconsistent. Some cohort studies report a significantly elevated risk (9, 10), while others show weak or non-significant associations (11, 12). This heterogeneity may arise from methodological differences, including variations in study design (e.g., cohort vs. cross-sectional), sample size, outcome event durations, follow-up duration, and the extent of adjustment for confounding factors such as cardiovascular risk and socioeconomic status (13). To address these inconsistencies, we conducted a systematic review and meta-analysis to provide a quantitative synthesis of the available evidence. The primary objective is to more precisely define the association between SLE and cognitive impairment or dementia, thereby offering robust evidence to inform clinical practice and guide future research.

## 2 Methods

This systematic review was conducted in accordance with the Preferred Reporting Items for Systematic Reviews and Meta-Analyses (PRISMA) 2020 statement (14) and was based on a prospectively registered protocol (PROSPERO: CRD420251148236).

### 2.1 Search strategy

We systematically searched PubMed, Embase, and the Cochrane Library for relevant literature published from their inception until September 12, 2025, to investigate the association between SLE and cognitive impairment or dementia. The search strategy combined Medical Subject Headings (MeSH) and free-text terms, primarily including “Systemic Lupus Erythematosus”, “Dementia”, “Cognitive Dysfunction”, and their related synonyms. Additionally, we manually screened the reference lists of relevant systematic review and included studies (15–17) to identify potentially eligible publications. The detailed search strategy is provided (**Supplementary Tables 1**–**3**).

### 2.2 Inclusion criteria

The inclusion criteria for studies followed the PICOS principle: (a) Population: Patients with a definitive diagnosis of systemic lupus erythematosus; (b) Exposure: Confirmed diagnosis of SLE [e.g., using American College of Rheumatology (ACR) classification criteria or the International Classification of Diseases (ICD) code]; (c) Comparator: Individuals without SLE, including healthy populations or patients with other chronic diseases (e.g., diabetes, hypertension, or osteoarthritis); (d) Outcome: Quantitative point estimates [odds ratios (ORs), hazard ratios (HRs), relative risks (RRs), or standardized incidence ratios (SIRs)] with their corresponding measures of variance [95% confidence intervals (CIs)] for the association between SLE and cognitive impairment, dementia, or Alzheimer’s disease; (e) Study Design: Observational studies, including cohort studies (prospective or retrospective), case-control studies, and cross-sectional studies.

### 2.3 Exclusion criteria

The exclusion criteria were as follows: (a) Reviews, commentaries, case reports, conference abstracts, and duplicate publications; (b) Studies without a control group or that did not report relevant outcome measures.

### 2.4 Literature screening

After importing the search results into NoteExpress software and removing duplicates, two investigators (YN Wang and S Jiang) independently performed the study selection. The screening process consisted of two stages: initially, titles and abstracts were reviewed to identify potentially eligible studies; subsequently, full texts were retrieved and assessed in detail for final inclusion determination based on the eligibility criteria. Any disagreements were resolved through discussion or by consultation with a third investigator (XL Li).

### 2.5 Data extraction

Prior to formal data extraction, the data extraction form was piloted using two studies to ensure consistency and clarity. Extracted data included: first author, publication year, country, study design, data source, sample size, participant characteristics, SLE diagnostic criteria, outcome definition, follow-up duration (for cohort studies), effect estimates (e.g., OR, HR, RR, SIR with their 95% confidence intervals), and covariates adjusted for in multivariable analysis.

### 2.6 Quality assessment

The methodological quality of the included cohort studies was assessed using the Newcastle-Ottawa Scale (NOS) (18). This scale covers three domains: selection of study groups, comparability of groups, and ascertainment of either the exposure or outcome. Studies with a total score of ≥ 7 stars were considered high quality. For cross-sectional studies, quality was assessed using the 11-item checklist recommended by the Agency for Healthcare Research and Quality (AHRQ) (19). Studies meeting ≥ 8 criteria were deemed high quality. The assessment was conducted independently by two investigators, and any inconsistencies were resolved through consensus discussion.

### 2.7 Statistical analysis

All statistical analyses were performed using Stata software (version 14.0). Given the limited number of included studies and the diversity of reported effect measures (including OR, HR, RR, SIR), these metrics were uniformly treated as approximations of the OR for pooling. This approach is based on the rare disease assumption, under which the odds ratio approximates the risk ratio when the outcome incidence is low (20, 21). A large-scale systematic review reported that the pooled incidence rate of dementia in community-dwelling adults aged 60+ was approximately 17 per 1000 person−years (22), well below the 10% threshold typically used to satisfy the rare disease assumption. More critically, the RR from cohort studies and the OR from cross-sectional studies are all valid measures of association that can be combined under the generic framework of an OR to provide a unified estimate across different study designs (23). If a study provided multiple effect estimates, the most fully adjusted value was extracted. The choice between a fixed-effect or random-effects model for meta-analysis was based on the observed degree of heterogeneity. Heterogeneity was evaluated using Cochran’s Q test (with a significance level of p < 0.10) and the I² statistic. For analyses where the I² value was below 50%, indicating low heterogeneity, the fixed-effect model was applied to provide more precise estimates. Conversely, for analyses with I² ≥ 50%, the random-effects model was retained (24, 25). To explore potential sources of heterogeneity, we conducted pre-specified subgroup analyses based on factors including: gender, study design, geographical region, and case group sample size. Furthermore, we performed sensitivity analyses by sequentially removing individual studies to examine the robustness of the pooled results. Publication bias was evaluated using funnel plots, Egger’s test, and Begg’s test, noting that the statistical power of these analyses is constrained by the limited number of included studies (26).

### 2.8 Patient and public involvement

Patients or the public were not involved in the design, conduct, reporting, or dissemination plans of this research because it is a meta-analysis based exclusively on previously published studies.

## 3 Results

### 3.1 Study selection

**Figure 1:**
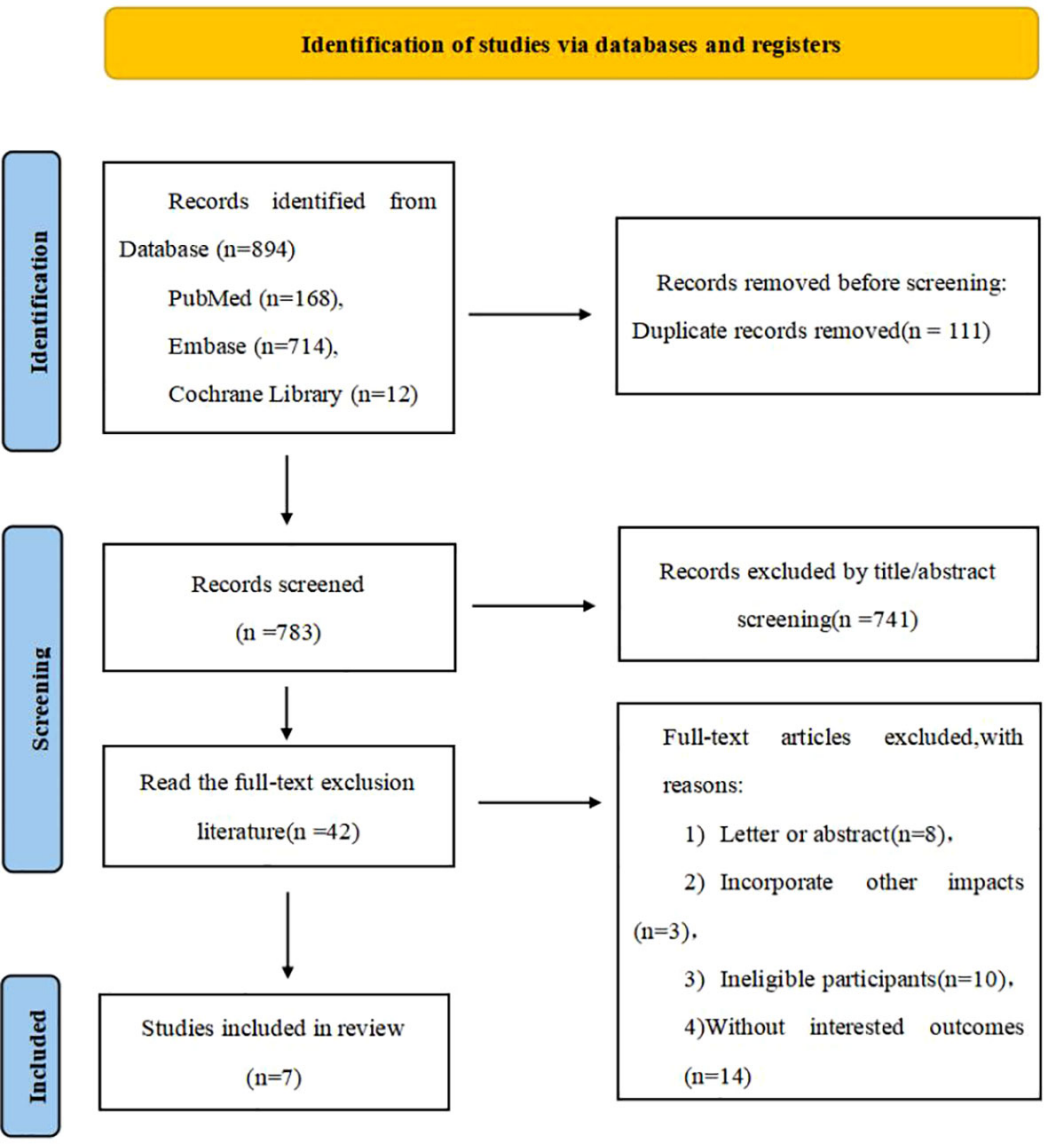
PRISMA flow chart of study selection.

The initial systematic search identified 894 records. After removing 111 duplicates, the titles and abstracts of 783 records were screened. Of these, 42 articles underwent full-text review. Ultimately, 7 studies (11, 27–32) met all the inclusion criteria and were included in the final meta-analysis. The detailed selection process is summarized in the PRISMA flow diagram (**Figure 1**). Our search strategy included terms for specific dementia subtypes (e.g., Alzheimer’s disease), and the retrieved studies predominantly reported data on the broader outcomes of “all-cause dementia” or “cognitive impairment.” Therefore, our primary analysis focused on this composite outcome of neurocognitive impairment. All included studies investigated the association with all-cause dementia or cognitive impairment.

### 3.2 Characteristics of included studies

The 7 studies were published between 2008 and 2025, with a total population exceeding 7 million participants. The sample size of SLE patients ranged from 60 to 5,371. Geographically, four studies (11, 28, 30, 31) were conducted in Asia (Taiwan and Israel), and the remaining three (27, 29, 32) were from Europe and Africa. Regarding study design, two were cross-sectional studies (27, 28), and five (11, 29–32) were retrospective cohort studies. The baseline characteristics of the included studies are summarized (**Table 1**).

**Table 1:** Characteristics of studies included in the meta-analysis.

Author	Year	Country	Study type	Sample size	Follow up	Age	Diagnosis of SLE	Diagnosis of result	Confounders adjusted
Ben Bouzid, A (27).	2025	Tunis	cross-sectional study	SLE:60, no SLE:40	/	SLE 42.6 ± 13.9; No-SLE 44.1 ± 14.4	ACR 1997	≥5/8 abnormal cognitive domains (assessed via neuropsychological battery)	Hypercholesterolemia, Sjögren’s syndrome, and marriage
Gendelman, O (28)	2018	Israel	cross‐sectional study	SLE:4886, no SLE:24,430	/	51.2 ± 16.5	Primary care physicians or from hospital records	Primary care physicians or from hospital records	Age, Gender, Diabetes, Hypertension, Hyperlipidemia
Lin, T. M (11)	2018	Taiwan	retrospective cohort	SLE:3062, no SLE:138,640	ARD: 5.97years; non-ARD:6.37years	59.80	ICD-9	ICD-9	age group, sex, and comorbidities
Wotton, C. J (29)	2017	UK	retrospective cohort	SLE: N/A, no SLE:700w	5.9years	52.3	ICD-10	ICD9; ICD10	sex, age in 5-year bands, time period in single calendar years, region of residence, and deprivation score associated with patients’ area of residence, in quintiles
Lin, Y. R (30)	2016	Taiwan	retrospective cohort	SLE:1,074, no SLE:5,370	7 years	/	ICD-9	ICD-9	age, sex, DM, hypertension, hyperlipidemia, coronary heart disease, Parkinson's disease, affective psychosis, and stroke
Lu, K (31).	2014	Taiwan	retrospective cohort	SLE:300, no SLE:6,105	5 years	/	ICD-9	ICD-9	demographic characteristics and selected comorbidities in patients
Sundquist, K (32).	2008	Sweden	retrospective cohort	SLE:5,371, no SLE: Entire Swedish population	31years	men 53; women 46	ICD-8; ICD-9; ICD-10	ICD-8; ICD-9; ICD-10	age, sex, time period, and geographic region

### 3.3 Risk of bias

The methodological quality of the cohort studies was assessed using the NOS, with scores ranging from 5 to 8 stars indicating a generally high quality. The cross-sectional studies, evaluated with the AHRQ checklist, demonstrated moderate to good quality. Overall, the methodological rigor of the included studies was acceptable (**Table 2**).

**Table 2:** Newcastle-Ottawa quality of cohort studies.

Author	Year	Selection	Comparability	Exposure	Overall quality score
Lin, T. M (11).	2018	★★★	★	★★	6
Wotton, C. J (29).	2017	★★	★	★★	5
Lin, Y. R (30).	2016	★★★★	★★	★★	8
Lu, K (31).	2014	★★★★	★★	★★	8
Sundquist, K (32).	2008	★★★★	★	★★	7

The ★ symbol represents one star in the Newcastle-Ottawa Scale; the total number of stars indicates study quality.

### 3.4 Association between SLE and cognitive impairment or dementia

#### 3.4.1 Meta-analysis results

**Figure 2:**
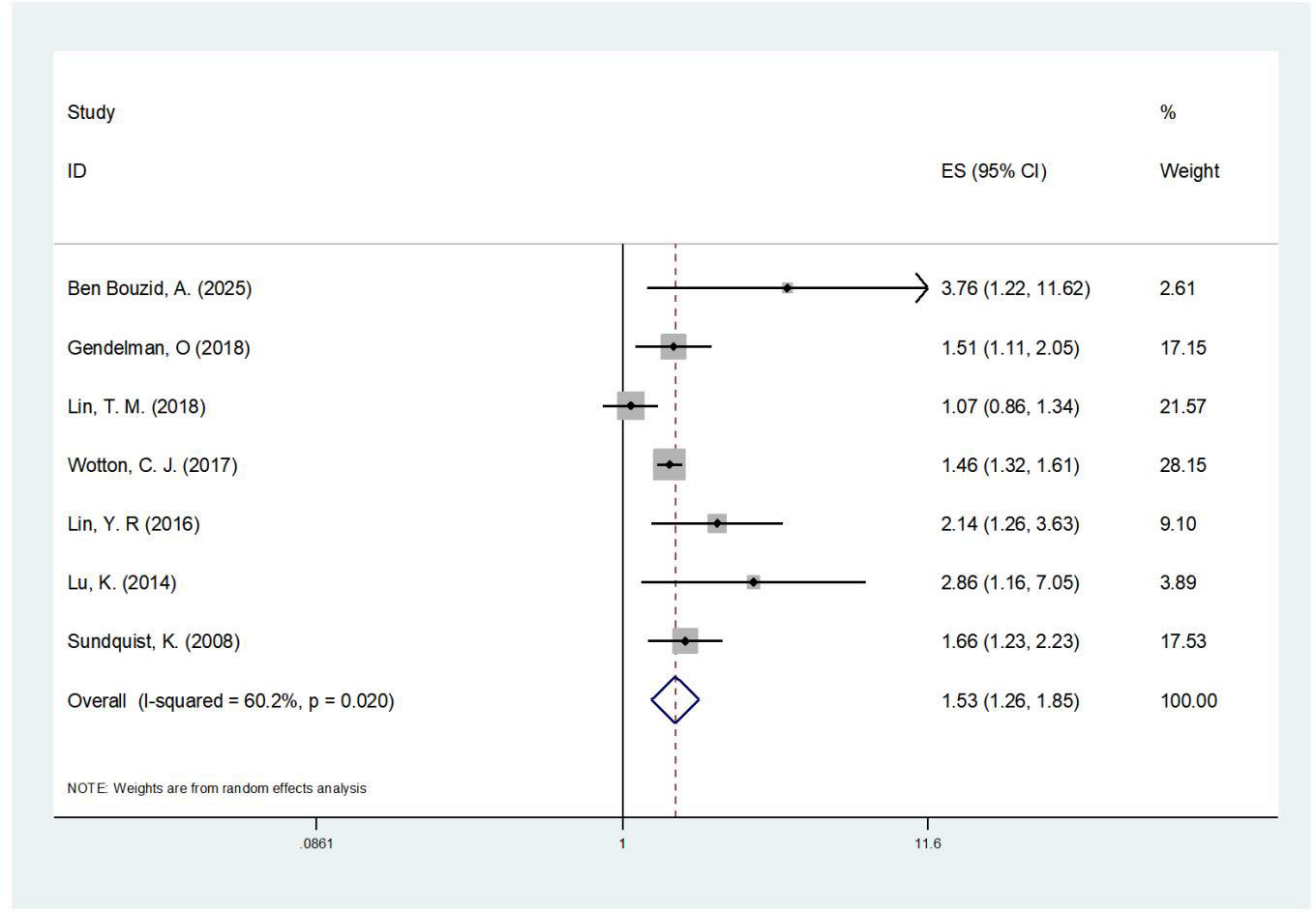
Forest plot for the association between SLE and cognitive impairment or dementia.

The random-effects meta-analysis revealed a significant association between SLE and cognitive impairment/dementia, with a pooled OR of 1.530 (95% CI: 1.264–1.851; p < 0.001) (**Figure 2**). This indicates a 53% higher odds of cognitive impairment or dementia in SLE patients compared to non-SLE populations. Owing to the considerable heterogeneity observed across studies (I² = 60.2%; p = 0.020), we conducted a leave-one-out sensitivity analysis. The results demonstrated that our overall finding was robust, as no individual study significantly influenced the pooled effect size.

#### 3.4.2 Subgroup analysis

To explore potential sources of heterogeneity, pre-specified subgroup analyses were performed (**Table 3**). Given the small number of studies in each subgroup, these findings should be interpreted as exploratory. The result showed that a significant association was observed in cohort studies (OR = 1.499, 95% CI: 1.198–1.875, I² = 67.2%). The point estimate was higher in cross-sectional studies (OR = 2.014) but was not statistically significant (95% CI: 0.877–4.622). Higher odds were also observed in both women (OR = 1.982, 95% CI: 1.249–3.146) and men (OR = 1.428, 95% CI: 1.132–1.802). Notably, no heterogeneity was found in the male subgroup (I² = 0.0%), whereas heterogeneity was substantial in the female subgroup (I² = 90.9%). It was consistently observed in both Asian (OR = 1.560, 95% CI: 1.076–2.262) and non-Asian populations (OR = 1.488, 95% CI: 1.355–1.635). Studies with fewer than 1000 SLE cases yielded a higher odds estimate (OR = 3.182) than those with 1000 or more cases (OR = 1.473), with both estimates being statistically significant.

**Table 3:** Results of subgroup analysis.

Subgroup	Included studies	OR		Heterogeneity	
		95% CI	P-values	I^2^	P-values
Study Type					
Retrospective cohort	5	1.499 (1.198-1.875)	0.000	67.2%	0.016
Cross-sectional study	2	2.014 (0.877-4.622)	0.099	57.3%	0.126
Gender					
Women	3	1.982 (1.249-3.146)	0.004	90.9%	0.000
Men	3	1.428 (1.132-1.802)	0.003	0.00%	0.474
Region					
Asia	4	1.560 (1.076-2.262)	0.019	70.6%	0.017
Non-Asia	3	1.488 (1.355-1.635)	0.000	38.4%	0.197
Case group					
<1000	2	3.182 (1.573-6.438)	0.001	0.0%	0.710
≥1000	4	1.473 (1.113-1.950)	0.007	68.4%	0.024

#### 3.4.3 Sensitivity analysis and publication bias

A leave-one-out sensitivity analysis demonstrated that the pooled effect size was not substantially altered by the removal of any single study, confirming the robustness of the findings (**Supplementary Figure 1**). Visual inspection of the funnel plot for the primary outcome suggested possible asymmetry (**Supplementary Figure 2**). Egger’s regression test was not statistically significant (p = 0.234>0.05). However, Begg’s test yielded a significant result (p = 0.024). Given the limited number of included studies, the power of these tests is low, and the results should be interpreted with caution. To further assess the potential impact of publication bias, the trim-and-fill method was applied. This analysis suggested that three studies might be missing (**Supplementary Figure 3**). After imputing these potentially missing studies, the pooled OR from the random-effects model was adjusted from 1.530 (95% CI: 1.264–1.851) to 1.395 (95% CI: 1.143–1.702). The fact that the adjusted estimate remained statistically significant confirms the robustness of our primary finding. Additionally, to assess the potential impact of outcome definition heterogeneity, we performed a sensitivity analysis excluding the only study that defined cognitive impairment using neuropsychological battery tests (27). The pooled OR for the remaining six studies (all reporting dementia or Alzheimer’s disease) was 1.48 (95% CI: 1.24–1.78, I² = 59.3%), which was consistent with the main analysis (**Supplementary Figure 4**).

## 4 Discussion

### 4.1 Main findings

This study systematically synthesizes data from 7 observational studies to assess the relationship between SLE and broader neurocognitive impairments, including dementia, Alzheimer’s disease, and cognitive dysfunction. Our analysis reveals a significant 53% higher odds (OR = 1.53) of neurocognitive impairment among SLE patients, offering robust evidence that SLE plays a substantial role in neuropsychiatric cognitive decline.

These findings align with a prior systematic review (15, 17), more importantly, through comprehensive subgroup analyses, we demonstrate that this elevated odds is consistent across different study designs (cohort studies), geographic regions (Asian and non-Asian populations), and genders. Notably, we identified that the association remains significant regardless of study size, though with a higher point estimate in smaller studies—an insight not systematically addressed in earlier syntheses. While previous meta-analyses (15, 17) have reported similar associations, our study offers several modest advances, including a substantially larger sample size, an updated literature search through September 2025, and an exploratory subgroup analysis by sample size that revealed a potential small-study effect. Additionally, we have attempted to integrate our epidemiological findings with recent Mendelian randomization and neuroimaging evidence to provide a broader mechanistic context. We hope these refinements strengthen the epidemiological foundation for considering SLE as a significant correlate of cognitive decline.

### 4.2 Interpretation of findings

The magnitude of association observed (pooled OR = 1.53) is biologically plausible and aligns with the prevailing hypothesis of chronic inflammation-driven neurodegeneration. The pathophysiological link between SLE and cognitive decline is likely multifactorial and progressive, forming a continuum from subtle cognitive impairment to overt dementia (4).

First, chronic neuroinflammation is central to these processes. Persistent pro-inflammatory activity in SLE patients can compromise the blood-brain barrier and activate microglia (33, 34), creating an environment conducive to synaptic dysfunction, which manifests as cognitive impairment, or, in more severe cases, irreversible neuronal degeneration leading to dementia (35). In particular, specific autoantibodies have been implicated in directly disrupting synaptic function. DNRAbs act as positive allosteric modulators of NMDARs, leading to excitotoxicity, microglia-mediated synaptic pruning, and memory impairment (4, 36). Anti-ribosomal P protein antibodies that cross-react with neuronal surface P antigen (NSPA) (5, 6) trigger calcium influx, impair glutamatergic receptor trafficking and recycling, and induce a microglial response associated with structural synaptic alterations (37), ultimately contributing to hippocampal-dependent memory deficits. Neuroimaging evidence of hippocampal atrophy and corpus callosum damage in SLE patients (4, 16, 35, 38) provides a direct structural correlate for this process, bridging reversible functional impairment with permanent structural change.

Second, cerebrovascular pathology plays a crucial role. SLE-associated vasculopathy and accelerated atherosclerosis (39) contribute to both macrovascular and microvascular disease, increasing the risk of stroke-related dementia and potentially causing chronic cerebral hypoperfusion leading to vascular cognitive impairment (38). Notably, among autoimmune diseases, SLE has been shown to carry a particularly high neurodegenerative risk due to its direct autoantibody-mediated attacks on the central nervous system and the induction of cerebrovascular pathology (39, 40), thereby supporting the results of this meta-analysis.

However, a recent Mendelian randomization study failed to establish a causal genetic link between SLE and dementia (41). It suggests that the strong association observed in epidemiological studies like ours may be primarily driven by acquired or state-dependent factors related to SLE disease activity and its treatment, rather than a shared genetic predisposition. Potential confounders along this pathway include the long-term use of corticosteroids (with their known neuropsychiatric side effects), the high burden of SLE-associated cardiovascular comorbidities, and the frequent co-occurrence of depression and anxiety (42). Thus, SLE may be associated with higher odds of dementia predominantly through the downstream consequences of chronic inflammation and vascular injury, rather than as a direct genetic consequence.

### 4.3 Clinical and research implications

The significant and consistent association between SLE and cognitive risk underscores an urgent need to integrate standardized cognitive screening into routine SLE management, particularly for high-risk subgroups such as women, patients with long disease duration or high inflammatory burden. Proactive management should follow, including optimization of SLE disease control, aggressive treatment of cardiovascular comorbidities, and addressing psychiatric co-morbidities. Concurrently, patients and clinicians require better education regarding this neuropsychiatric risk to facilitate early reporting and intervention. Future research must prioritize prospective longitudinal studies with uniform neuropsychological assessments to clarify the trajectory of decline, explore biomarkers for early detection, and ultimately develop targeted interventions to mitigate this debilitating complication.

### 4.4 Implications and limitations

The main strengths of this study include adherence to PRISMA guidelines, rigorous subgroup and sensitivity analyses, and the overall robustness of our findings. By integrating multiple neurocognitive outcomes, we have provided a comprehensive assessment of the long-term neuropsychiatric associations with SLE.

However, several limitations must be acknowledged. First, given the limited number of studies included, the subgroup analyses may lack sufficient statistical power and should not be overinterpreted as reflecting true differences between populations. Future high-quality prospective studies are needed to further validate these exploratory findings. Second, the inherent heterogeneity in both outcome definitions and effect measures across studies may contribute to the observed statistical heterogeneity. Only one included study (27) used standardized neuropsychological battery tests to define cognitive impairment; the remaining studies relied on ICD-based diagnostic codes for dementia or cognitive dysfunction. This heterogeneity in outcome ascertainment—particularly the lack of uniform thresholds for defining cognitive impairment—may contribute to the observed statistical heterogeneity and should be addressed in future studies using standardized assessment tools. Third, as with all meta-analyses of observational studies, the demonstrated association does not imply causality. Residual confounding and other biases cannot be fully excluded, and the findings should be interpreted as reflecting an observational association rather than a causal effect. Finally, although Egger’s test did not indicate substantial publication bias, the trim-and-fill method suggested a slight potential for bias, which did not alter the overall conclusions after adjustment.

In summary, building upon existing knowledge, this meta-analysis enhances the reliability of the SLE-neurocognitive impairment link through updated data, more comprehensive subgroup analyses, and by providing clinical correlation to neuroimaging discoveries. This underscores the necessity of integrating cognitive assessment and early intervention into the routine clinical management of SLE.

## 5 Conclusions

This meta-analysis highlights that SLE is associated with significantly higher odds of neurocognitive impairment, urging clinicians to incorporate regular cognitive screenings in SLE patient care. Future research should focus on long-term studies with standardized neuropsychological tools to better understand cognitive decline and identify key predictors.

## Data Availability

The original contributions presented in the study are included in the article/**Supplementary Material**. Further inquiries can be directed to the corresponding authors.

## Supplementary material

Supplementary Figure 1 Sensitivity analysis for 7 studies included in the meta-analysis.

Supplementary Figure 2 Funnel plot for 7 studies included in the meta-analysis.

Supplementary Figure 3 Funnel plot for dementia or cognitive impairment in SLE patients.

Supplementary Figure 4 Forest plot of the sensitivity analysis excluding the cognitive impairment study.
