# Correction: The association between systemic lupus erythematosus and cognitive impairment or dementia: a meta-analysis

**DOI:** 10.3389/fimmu.2026.1902326

**Published:** 2026-06-17

**Authors:** Yanan Wang, Sai Jiang, Yicong Liu, Hejing Pan, Lin Huang, Chengping Wen, Xuanlin Li, Zhijun Xie

**Affiliations:** 1 College of Basic Medical Science, Zhejiang Chinese Medical University, Hangzhou, China; 2 Zhejiang Chinese Medical University Jinhua Research Institute, Jinhua, China

**Keywords:** cognitive impairment, dementia, meta-analysis, neuropsychiatric manifestations, systemic lupus erythematosus

There was a mistake in **Table 3** as published. In the “Case group” subgroup, the study by Wotton 2017 (reference 29) was erroneously included in the <1000 group. However, this study did not report the total number of SLE patients and therefore should have been excluded from the sample size subgroup analysis. As a result, the <1000 group now contains 2 studies, and the ≥1000 group now contains 4 studies. The corrected **Table 3** appears below.

**Table 3:** Results of subgroup analysis.

Subgroup	Included studies	OR		Heterogeneity	
		95%CI	P-values	I^2^	P-values
Study type					
Retrospective cohort	5	1.499(1.198-1.875)	0.000	67.2%	0.016
Cross-sectional study	2	2.014(0.877-4.622)	0.099	57.3%	0.126
Gender					
Women	3	1.982(1.249-3.146)	0.004	90.9%	0.000
Men	3	1.428(1.132-1.802)	0.003	0.0%	0.474
Region					
Asia	4	1.560(1.076-2.262)	0.019	70.6%	0.017
Non-Asia	3	1.488(1.355-1.635)	0.000	38.4%	0.197
Case group					
<1000	2	3.182(1.573-6.438)	0.001	0.0%	0.710
≥1000	4	1.473(1.113-1.950)	0.007	68.4%	0.024

In the section **Results**, *3.4.2 Subgroup analysis*, the following sentence was based on the incorrect **Table 3** and therefore contained an error: “Studies with fewer than 1000 SLE cases yielded a higher odds estimate (OR = 2.087) than those with 1000 or more cases (OR = 1.473), with both estimates being statistically significant.” The sentence has now been corrected to read: “Studies with fewer than 1000 SLE cases yielded a higher odds estimate (OR = 3.182) than those with 1000 or more cases (OR = 1.473), with both estimates being statistically significant.”

The original version of this article has been updated.

