# Supplementary figures and images for: The association between systemic lupus erythematosus and cognitive impairment or dementia: a meta-analysis
Source: Front Immunol. 2026 Jun 2;17:1795410. doi: 10.3389/fimmu.2026.1795410 (PMC13269413; doi:10.3389/fimmu.2026.1795410)

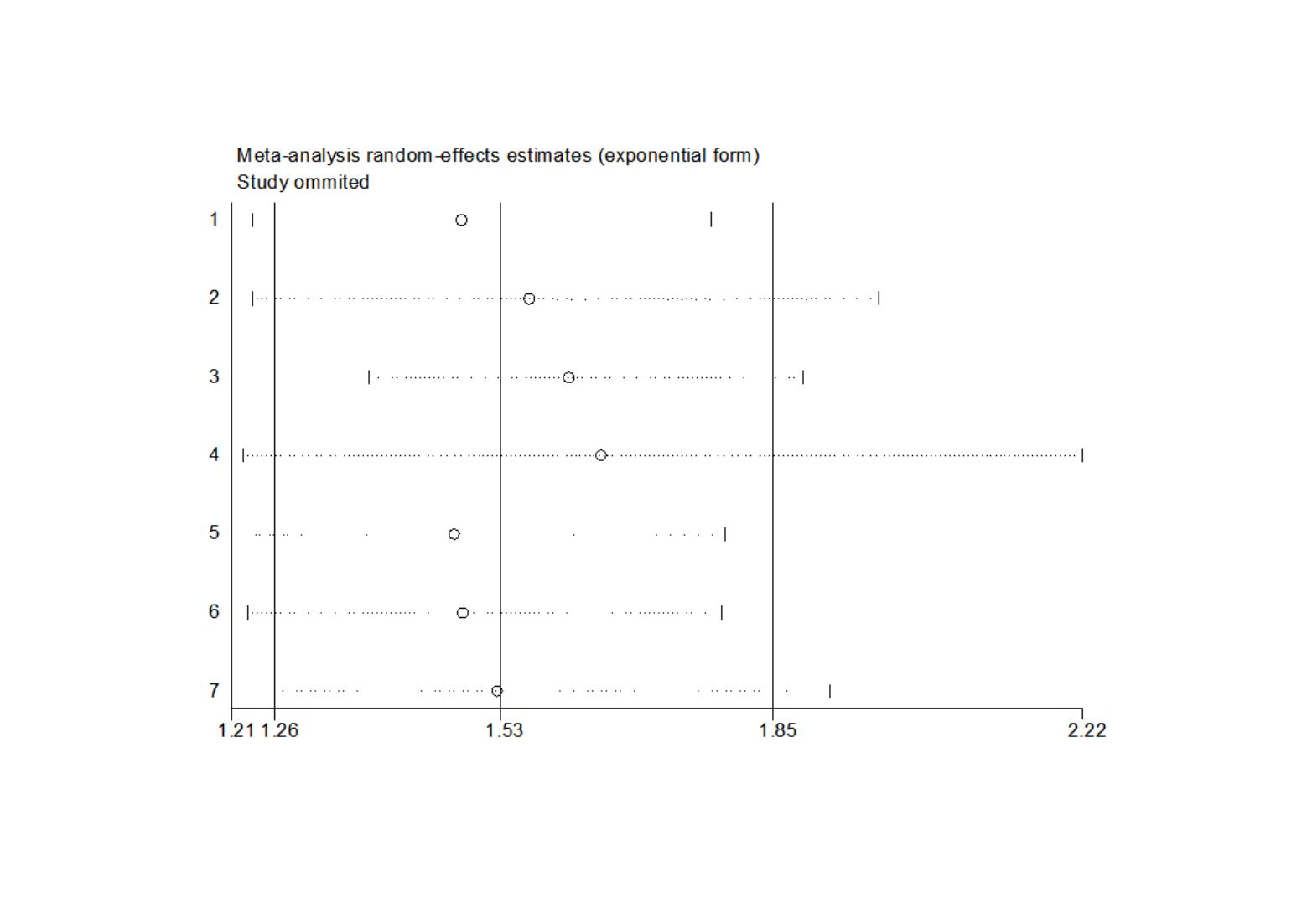

Supplement: Supplementary Figure 1 — Sensitivity analysis for 7 studies included in the meta-analysis. [file Image1.jpeg]

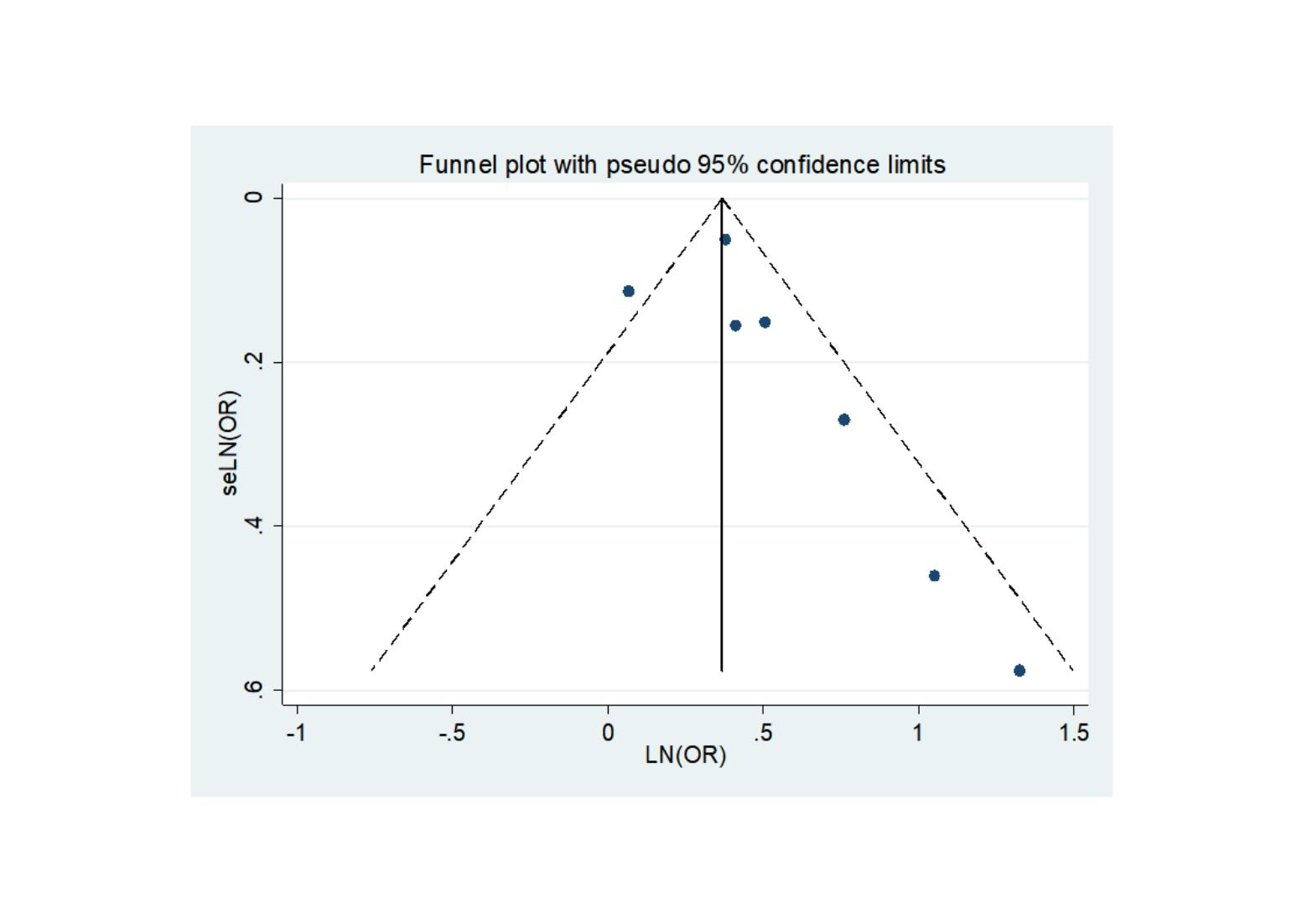

Supplement: Supplementary Figure 2 — Funnel plot for 7 studies included in the meta-analysis. [file Image2.jpeg]

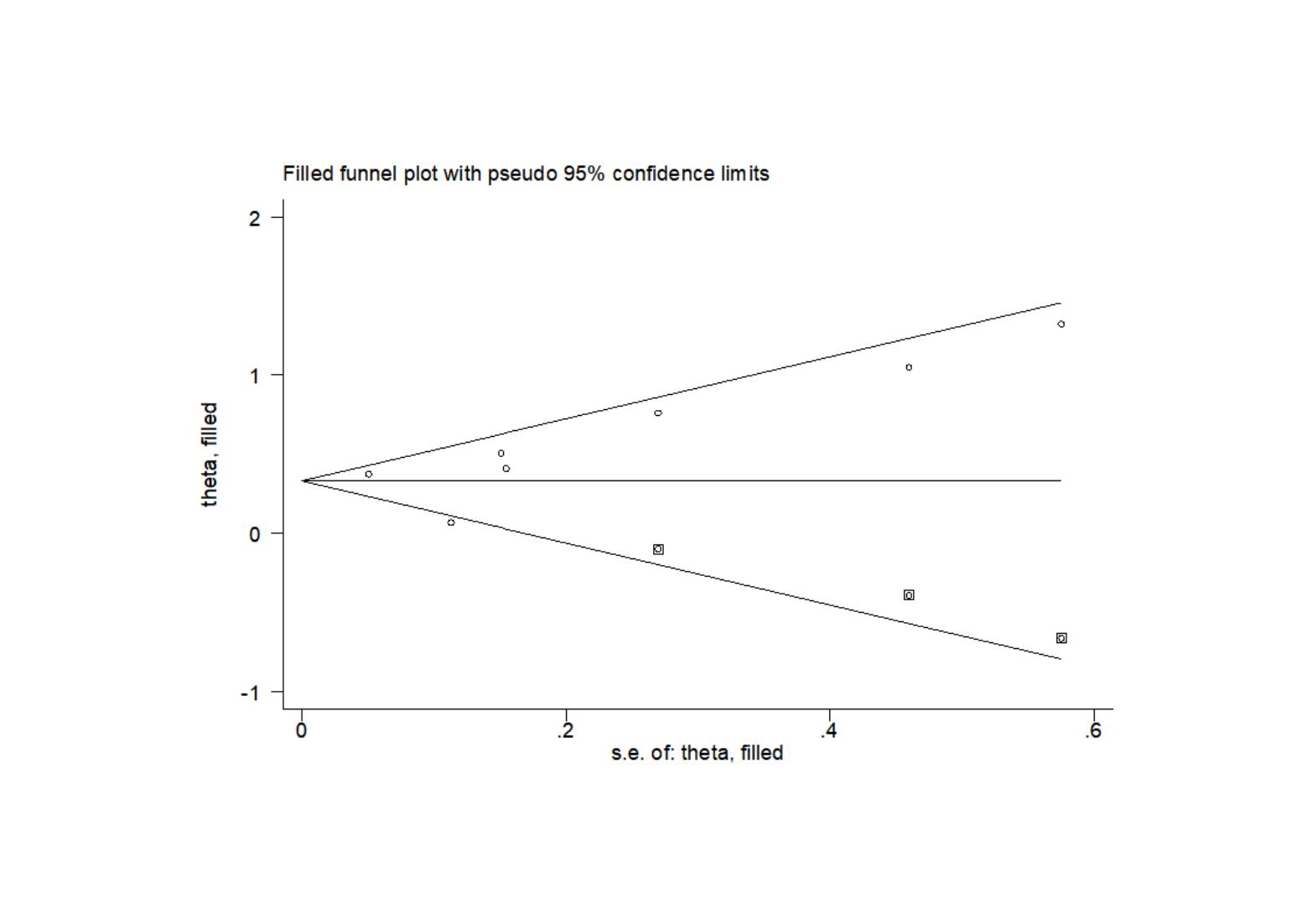

Supplement: Supplementary Figure 3 — Funnel plot for dementia or cognitive impairment in SLE patients. [file Image3.jpeg]

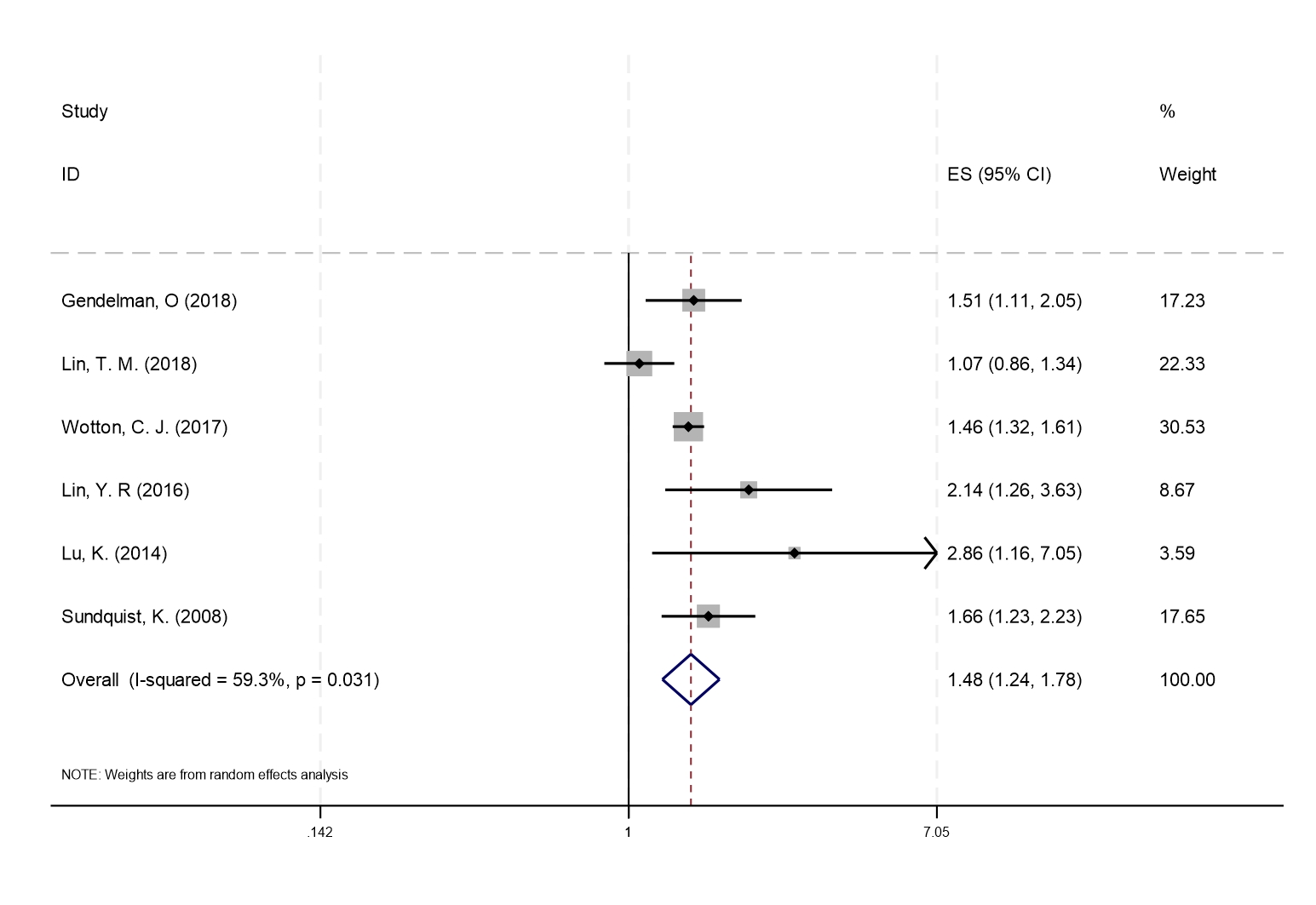

Supplement: Supplementary Figure 4 — Forest plot of the sensitivity analysis excluding the cognitive impairment study. [file Image4.jpeg]
