# Supplementary material for: The association between systemic lupus erythematosus and cognitive impairment or dementia: a meta-analysis
Source: Front Immunol. 2026 Jun 2;17:1795410. doi: 10.3389/fimmu.2026.1795410 (PMC13269413; doi:10.3389/fimmu.2026.1795410)
Supplement: Supplementary file 5 [file DataSheet1.pdf]

Supplementary Tables 1-3

Supplementary Table 1 PubMed

No.	Content	Result
#1	Search: "Lupus Erythematosus, Systemic"[Mesh] Sort by: Most Recent	71,446
#2	Search:(((systemic lupus erythemato*[Title/Abstract]) OR (Lupus Erythematosus Disseminatus[Title/Abstract])) OR (disseminated lupus erythemato*[Title/Abstract])) OR (lupus erythematosus visceralis[Title/Abstract]) Sort by: Most Recent	68,057
#3	Search: ("Lupus Erythematosus, Systemic"[Mesh]) OR ((((systemic lupus erythemato*[Title/Abstract]) OR (Lupus Erythematosus Disseminatus[Title/Abstract])) OR (disseminated lupus erythemato*[Title/Abstract])) OR (lupus erythematosus visceralis[Title/Abstract]))Sort by: Most Recent	90,734
#4	Search: "Cognition Disorders"[Mesh] Sort by: Most Recent	131,125
#5	Search:(((((((((cognition disorder*[Title/Abstract]) OR (cognitive complaint*[Title/Abstract])) OR (cognitive decline*[Title/Abstract])) OR (cognitive defic*[Title/Abstract])) OR (cognitive disabilit*[Title/Abstract])) OR (cognitive disturbance*[Title/Abstract])) OR (cognitive dysfunction*[Title/Abstract])) OR (cognitive impairment*[Title/Abstract])) OR (cognitive problem*[Title/Abstract])) OR (cognitive loss[Title/Abstract])Sort by: Most Recent	187,536
#6	Search: ("Cognition Disorders"[Mesh]) OR ((((((((((cognition disorder*[Title/Abstract]) OR (cognitive complaint*[Title/Abstract])) OR (cognitive decline*[Title/Abstract])) OR (cognitive defic*[Title/Abstract])) OR (cognitive disabilit*[Title/Abstract])) OR (cognitive	242,618

	disturbance*[Title/Abstract])) OR (cognitive dysfunction*[Title/Abstract])) OR (cognitive impairment*[Title/Abstract])) OR (cognitive problem*[Title/Abstract])) OR (cognitive loss[Title/Abstract]))Sort by: Most Recent	
#7	Search: "Alzheimer Disease"[Mesh] Sort by: Most Recent	135,742
#8	Search: Alzheimer*[Title/Abstract] Sort by: Most Recent	223,898
#9	Search: ("Alzheimer Disease"[Mesh]) OR (Alzheimer*[Title/Abstract])Sort by: Most Recent	236,990
#10	Search: "Dementia"[Mesh] Sort by: Most Recent	230,737
#11	Search: (((Dementi*[Title/Abstract]) OR (Amentia*[Title/Abstract])) OR (Senile Paranoid Dementia*[Title/Abstract])) OR (Familial Dementia*[Title/Abstract]) Sort by: Most Recent	169,436
#12	Search: ("Dementia"[Mesh]) OR ((((Dementi*[Title/Abstract]) OR (Amentia*[Title/Abstract])) OR (Senile Paranoid Dementia*[Title/Abstract])) OR (Familial Dementia*[Title/Abstract]))Sort by: Most Recent	298,213
#13	Search: ((("Cognition Disorders"[Mesh]) OR ((((((((((cognition disorder*[Title/Abstract]) OR (cognitive complaint*[Title/Abstract])) OR (cognitive decline*[Title/Abstract])) OR (cognitive defic*[Title/Abstract])) OR (cognitive disabilit*[Title/Abstract])) OR (cognitive disturbance*[Title/Abstract])) OR (cognitive dysfunction*[Title/Abstract])) OR (cognitive impairment*[Title/Abstract])) OR (cognitive problem*[Title/Abstract])) OR (cognitive loss[Title/Abstract]))) OR (("Alzheimer Disease"[Mesh]) OR (Alzheimer*[Title/Abstract]))) OR (("Dementia"[Mesh]) OR ((((Dementi*[Title/Abstract]) OR	515,186

	(Amentia*[Title/Abstract])) OR (Senile Paranoid Dementia*[Title/Abstract])) OR (Familial Dementia*[Title/Abstract])))Sort by: Most Recent	
#14	Search: (((("Cognition Disorders"[Mesh]) OR (((((((((cognition disorder*[Title/Abstract]) OR (cognitive complaint*[Title/Abstract])) OR (cognitive decline*[Title/Abstract])) OR (cognitive defic*[Title/Abstract])) OR (cognitive disabilit*[Title/Abstract])) OR (cognitive disturbance*[Title/Abstract])) OR (cognitive dysfunction*[Title/Abstract])) OR (cognitive impairment*[Title/Abstract])) OR (cognitive problem*[Title/Abstract])) OR (cognitive loss[Title/Abstract]))) OR (("Alzheimer Disease"[Mesh]) OR (Alzheimer*[Title/Abstract]))) OR (("Dementia"[Mesh]) OR ((((Dementi*[Title/Abstract]) OR (Amentia*[Title/Abstract])) OR (Senile Paranoid Dementia*[Title/Abstract])) OR (Familial Dementia*[Title/Abstract])))) AND (("Lupus Erythematosus, Systemic"[Mesh]) OR ((((systemic lupus erythemato*[Title/Abstract]) OR (Lupus Erythematosus Disseminatus[Title/Abstract])) OR (disseminated lupus erythemato*[Title/Abstract])) OR (lupus erythematosus visceralis[Title/Abstract])))Sort by: Most Recent	1,032
#15	Search: "Risk"[Mesh] Sort by: Most Recent	1,493,392
#16	Search: (risk*[Title/Abstract]) OR (relative risk*[Title/Abstract])Sort by: Most Recent	3,455,571
#17	Search: ("Risk"[Mesh]) OR ((risk*[Title/Abstract]) OR (relative risk*[Title/Abstract]))Sort by: Most Recent	3,962,413
#18	Search: ((((("Cognition Disorders"[Mesh]) OR (((((((((cognition disorder*[Title/Abstract]) OR (cognitive	168

	complaint*[Title/Abstract])) OR (cognitive decline*[Title/Abstract])) OR (cognitive defic*[Title/Abstract])) OR (cognitive disabilit*[Title/Abstract])) OR (cognitive disturbance*[Title/Abstract])) OR (cognitive dysfunction*[Title/Abstract])) OR (cognitive impairment*[Title/Abstract])) OR (cognitive problem*[Title/Abstract])) OR (cognitive loss[Title/Abstract]))) OR (("Alzheimer Disease"[Mesh]) OR (Alzheimer*[Title/Abstract]))) OR (("Dementia"[Mesh]) OR ((((Dementi*[Title/Abstract]) OR (Amentia*[Title/Abstract])) OR (Senile Paranoid Dementia*[Title/Abstract])) OR (Familial Dementia*[Title/Abstract])))) AND (("Lupus Erythematosus, Systemic"[Mesh]) OR ((((systemic lupus erythemato*[Title/Abstract]) OR (Lupus Erythematosus Disseminatus[Title/Abstract])) OR (disseminated lupus erythemato*[Title/Abstract])) OR (lupus erythematosus visceralis[Title/Abstract])))) AND (("Risk"[Mesh]) OR ((risk*[Title/Abstract]) OR (relative risk*[Title/Abstract])))Sort by: Most Recent	

Supplementary Table 2 Embase

No.	Content	Result
#1	'systemic lupus erythematosus'/exp	148,647
#2	'systemic lupus erythemato*':ab,ti OR 'lupus erythematosus disseminatus':ab,ti OR 'disseminated lupus erythemato*':ab,ti OR 'lupus erythematosus visceralis':ab,ti	99,157
#3	#1 OR #2	157,143
#4	'cognitive defect'/exp	733,468
#5	'cognition disorder*':ab,ti OR 'cognitive complaint*':ab,ti	274,417

	OR 'cognitive decline*':ab,ti OR 'cognitive defic*':ab,ti OR 'cognitive disabilit*':ab,ti OR 'cognitive disturbance*':ab,ti OR 'cognitive dysfunction*':ab,ti OR 'cognitive impairment*':ab,ti OR 'cognitive problem*':ab,ti OR 'cognitive loss':ab,ti	
#6	#4 OR #5	784,619
#7	'alzheimer disease'/exp	291,241
#8	alzheimer*:ab,ti	289,240
#9	#7 OR #8	345,978
#10	'dementia'/exp	524,074
#11	dementi*:ab,ti OR amentia*:ab,ti OR 'senile paranoid dementia*':ab,ti OR 'familial dementia*':ab,ti	234,487
#12	#10 OR #11	559,705
#13	#6 OR #9 OR #12	838,954
#14	#3 AND #13	3,503
#15	'risk'/exp	3,551,642
#16	risk*:ab,ti OR 'relative risk*':ab,ti	5,025,856
#17	#15 OR #16	5,972,373
#18	#14 AND #17	714

Supplementary Table 3 Cochrane Library

No.	Content	Result
#1	MeSH descriptor: [Lupus Erythematosus, Systemic] explode all trees	1,667
#2	(systemic lupus erythemato*):ti,ab,kw OR (Lupus Erythematosus Disseminatus):ti,ab,kw OR (disseminated lupus erythemato*):ti,ab,kw OR (lupus erythematosus visceralis):ti,ab,kw	3,366
#3	#1 or #2	3,605
#4	MeSH descriptor: [Cognition Disorders] explode all trees	9,065
#5	(cognition disorder*):ti,ab,kw OR (cognitive complaint*):ti,ab,kw OR (cognitive decline*):ti,ab,kw OR (cognitive defic*):ti,ab,kw OR	34,305

	(cognitive disabilit*):ti,ab,kw	
#6	(cognitive disturbance*):ti,ab,kw OR (cognitive dysfunction*):ti,ab,kw OR (cognitive impairment*):ti,ab,kw OR (cognitive problem*):ti,ab,kw OR (cognitive loss):ti,ab,kw	43,178
#7	#5 OR #6	61,170
#8	#4 OR #7	61,510
#9	MeSH descriptor: [Alzheimer Disease] explode all trees	5,698
#10	(Alzheimer*):ti,ab,kw	15,519
#11	#9 OR #10	15,519
#12	MeSH descriptor: [Dementia] explode all trees	10,100
#13	(Dementi*):ti,ab,kw OR (Amentia*):ti,ab,kw OR (Senile Paranoid Dementia*):ti,ab,kw OR (Familial Dementia*):ti,ab,kw	19,312
#14	#12 OR #13	22,821
#15	#8 OR #11 OR #14	78,511
#16	#3 AND #15	50
#17	MeSH descriptor: [Risk] explode all trees	55,991
#18	(risk*):ti,ab,kw OR (relative risk*):ti,ab,kw	336,656
#19	#17 OR #18	340,470
#20	#16 AND #19	12
